# The inflection point: a torque reference for lingual bracket positioning on the palatal surface curvature of the maxillary central incisor

**DOI:** 10.1186/s40510-018-0234-0

**Published:** 2018-10-08

**Authors:** Abdel Hadi Kanj, Joseph Bouserhal, Essam Osman, Ahmed Abdel Moneim El Sayed

**Affiliations:** 1 Department of Developmental Sciences, Division of Orthodontics, Faculty of Dentistry, Beirut Arab University (BAU), Beirut Campus, Main Building, 1st Floor, Tareek El Jadida P.O. Box 11-5020 Riad El Solh 11072809, Beirut, Lebanon; 2 Department of Orthodontics and Dentofacial Orthopedics, Henry M. Goldman School of Dental Medicine, Boston University, Boston, USA; 3 Department of Orthodontics, School of Dental Medicine, Saint Joseph University of Beirut, Beirut, Lebanon; 4 Department of Oral Rehabilitation Sciences, Division of Dental Biomaterials, Faculty of Dentistry, Beirut Arab University (BAU), Beirut, Lebanon

**Keywords:** Bracket positioning, Lingual orthodontics, Maxillary central incisor, Torque

## Abstract

**Background:**

Contrary to buccal orthodontics, lingual orthodontics has no reference for vertical bracket positioning on the maxillary central incisor. The aim of this study was to provide a reference point in relation to torque for lingual bracket positioning on the palatal surface curvature (PSC) of the maxillary central incisor.

**Methods:**

Cone beam computed tomography (CBCT) radiographs of 50 right maxillary central incisors from archives of a dental radiographic center were transferred to Photoshop, where their PSC was traced using pen-tool. The PSC torque angle values of the incisors were calculated in Excel using cubic poly-Bezier curves at 0.5-mm increments and at the inflection point of PSC. Descriptive statistics for the torque angle values of the increments and for the inflection point for the 50 incisors were then calculated. One-way ANOVA test was used to detect systematic differences between the increments, and Tukey test was used post-hoc.

**Results:**

For all incisors, increments incisal to inflection point exhibited progressive decrease in torque angle values from the first-calculated increment to inflection point while increments cervical to inflection point exhibited progressive increase from inflection point to last-calculated increment. Mean torque angle values of all the increments and inflection point showed high standard deviations and vast range of values. One-way ANOVA test was highly statistically significant (*p* < 0.0001) and most pairwise comparisons of the increments using Tukey test were significant.

**Conclusions:**

Inflection point can be used as a reference for bracket positioning on PSC. Cervically oriented shifts in vertical bracket position cause crown labial/root palatal movement cervical to inflection point and crown palatal/root labial movement incisal to it. A scientific mathematical justification for customized bracket torque prescriptions on PSC of maxillary central incisor was also provided.

**Electronic supplementary material:**

The online version of this article (10.1186/s40510-018-0234-0) contains supplementary material, which is available to authorized users.

## Background

The torque of a certain crown site can be assessed by viewing the proximal tooth aspect and then determining the inclination of the tangent at that site [1–13]. Consequently, each bracket site on a tooth will have an associated torque angle value (${Torque}_{Value}^{Angle}$) determined by the tangent’s inclination at that site. The difference in torque between two bracket positions can be found by subtracting their associated ${Torque}_{Values}^{Angle}$ [6–13]. The effect of vertical bracket position on torque in labial/buccal orthodontics has been discussed thoroughly in the literature [6–13] compared to a single study in lingual orthodontics [2]. Yet that lingual study used only four different vertical bracket positions to investigate that effect instead of using 0.5 mm or 1 mm tooth increments, akin to the studies of the labial/buccal orthodontics [6–8, 10–13]. In their turn, Kurz et al. [1] calculated the ${Torque}_{Value}^{Angle}$ of only one bracket site on the lingual surfaces of a set of maxillary and mandibular dentition, while Bryant et al. [4] with a mathematical equation of a parametric survival model could only calculate the maximum slope found at the inflection point of the palatal surface curvature (PSC) of the maxillary central incisor. The inflection point of a mathematical function is the point where the curvature of that function changes from convex to concave or vice versa [14].

${Torque}_{Value}^{Angle}$ calculation is generally accomplished by drawing tangents directly on the crown [8, 9]. Miethke pointed out that this method “is more or less subjective depending on the crown curvature” [8]. The error in angle measurement which can occur upon the use of drawn tangents to assess lingual surface curvature can exceed 4° [2], which calls for more accurate mathematical methods of angle measurement. The pen-tool in Adobe Photoshop Creative Cloud 2013 (Adobe Systems Inc., San Francisco, CA) can create cubic Bezier curves, which are parametric mathematical equations where the tangent at any point on these curves could be calculated by using the curve’s first derivative [15]. A cubic Bezier curve is formed by four control points and mathematically it is represented by two equations [16]. The two equations could be found by substituting the coordinates of the four control points in the mathematical formula of the cubic Bezier curve [16]. The initial and terminal control points of the cubic Bezier curve lie on the curve and are always its endpoints while the other two intermediate control points which determine its curvature do not generally lie on the curve [16].

Contrary to lingual orthodontics, conventional labial orthodontics has a reference point for bracket positioning reflected in the long axis point [17]. As there is no reference in lingual orthodontics for bracket positioning on PSC, the aim of this study was to find if the inflection point of PSC can be used as a torque reference for lingual bracket positioning (the inflection point of PSC is the anatomical landmark where the intersection between the convex and concave portions of PSC occurs). The use of cone beam computed tomography (CBCT) to calculate the ${Torque}_{Values}^{Angle}$ on the labial and buccal surface curvatures were made previously in two studies [12, 13]. To our knowledge, no study has been done using CBCT to assess the PSC of the maxillary central incisor through cubic poly-Bezier curves in Photoshop.

## Methods

Ethical committee approval was obtained from the university’s ethical board before beginning the study (pre-approval code: 2016H-0040-D-M-0155*).* To fulfill the aim of the study, 50 right maxillary central incisors (RMCI) were selected from CBCT archives of a dental radiographic center and then the ${Torque}_{Values}^{Angle}$ of their Photoshop-traced PSC were calculated at 0.5-mm increments and at the inflection point using the first derivative of their cubic poly-Bezier curve. A total of 50 CBCT radiographs containing both jaws were selected randomly from the archives of a radiographic center in a private office. Those radiographs were made for non-orthodontic reasons and were taken by a Kodak 9500C Cone Beam 3D machine (Kodak Dental Systems, Carestream Health Inc., Rochester, NY) at 10 mA, 80 KV and an exposure time of 10.8 s with a voxel dimension of 300 μm. The inclusion/exclusion criteria for the selection of each CBCT radiograph is detailed below.

### Inclusion criteria

- Radiographs should belong to individuals aged between 15 and 30.
- The absence of inter-incisal contact on the palatal surface of the RMCI on radiograph. Contact of the lower incisors on the PSC of the RMCI would not allow proper tracing of PSC.
- RMCI presenting palatal surface and incisal edge integrity on radiograph.

### Exclusion criteria

- Intra-oral presence of metal or amalgam restorations shown in radiograph.
- Intra-oral presence of labial or lingual brackets in radiograph.
- RMCI with attrition or caries or a dilacerated root in radiograph.

**Fig. 1:**
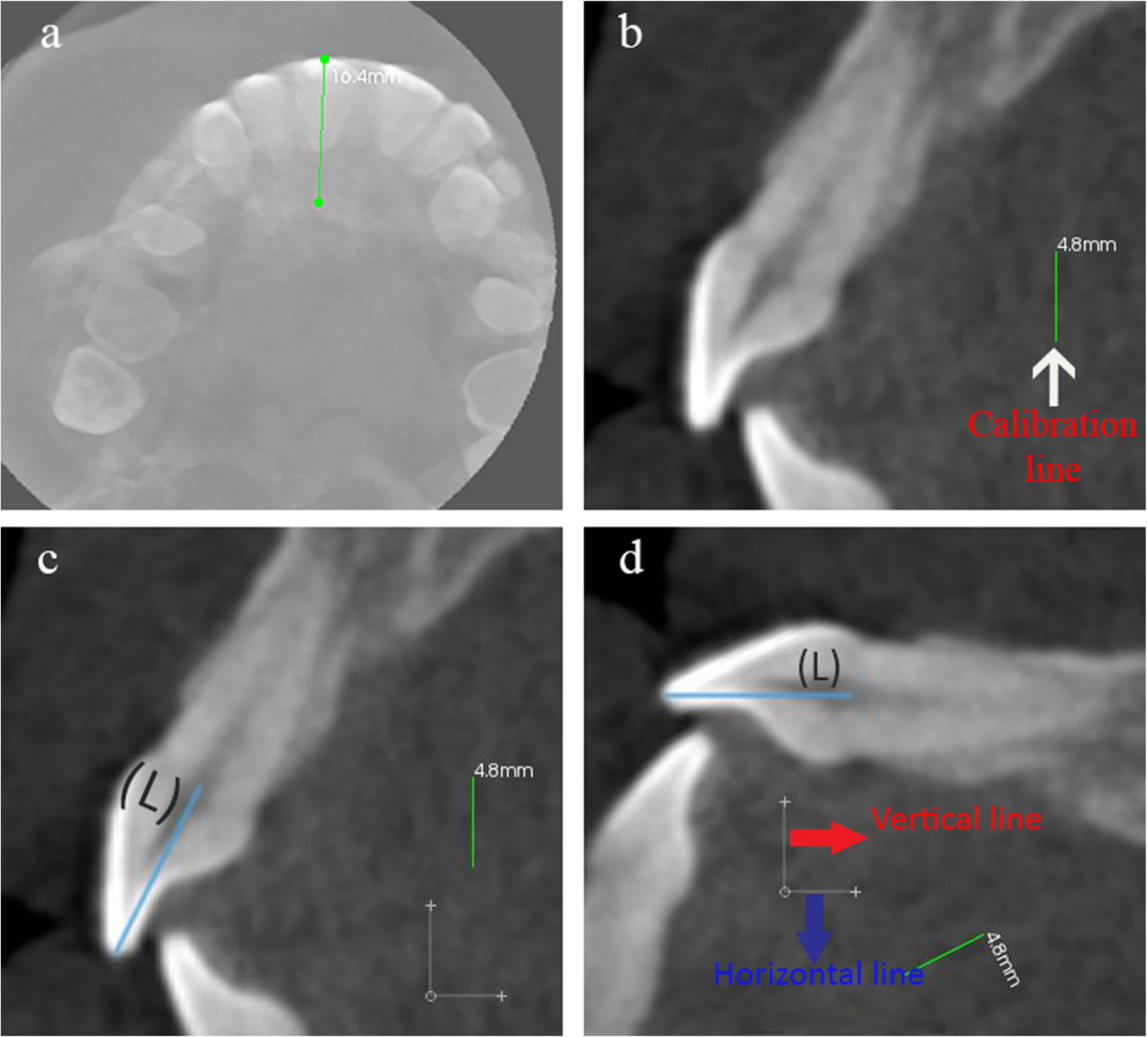
**a** Labio-palatal axis of RMCI (drawn line) was crossed by the sagittal plane indicator in the axial slice of CBCT image. **b** Sagittal slice of CBCT image showing proximal aspect of RMCI and calibration line. **c** The red line joins palatal and labial CEJs, while blue line (L) represents crown’s long axis. **d** Image rotation was done such that line (L) became horizontal

**Fig. 2:**
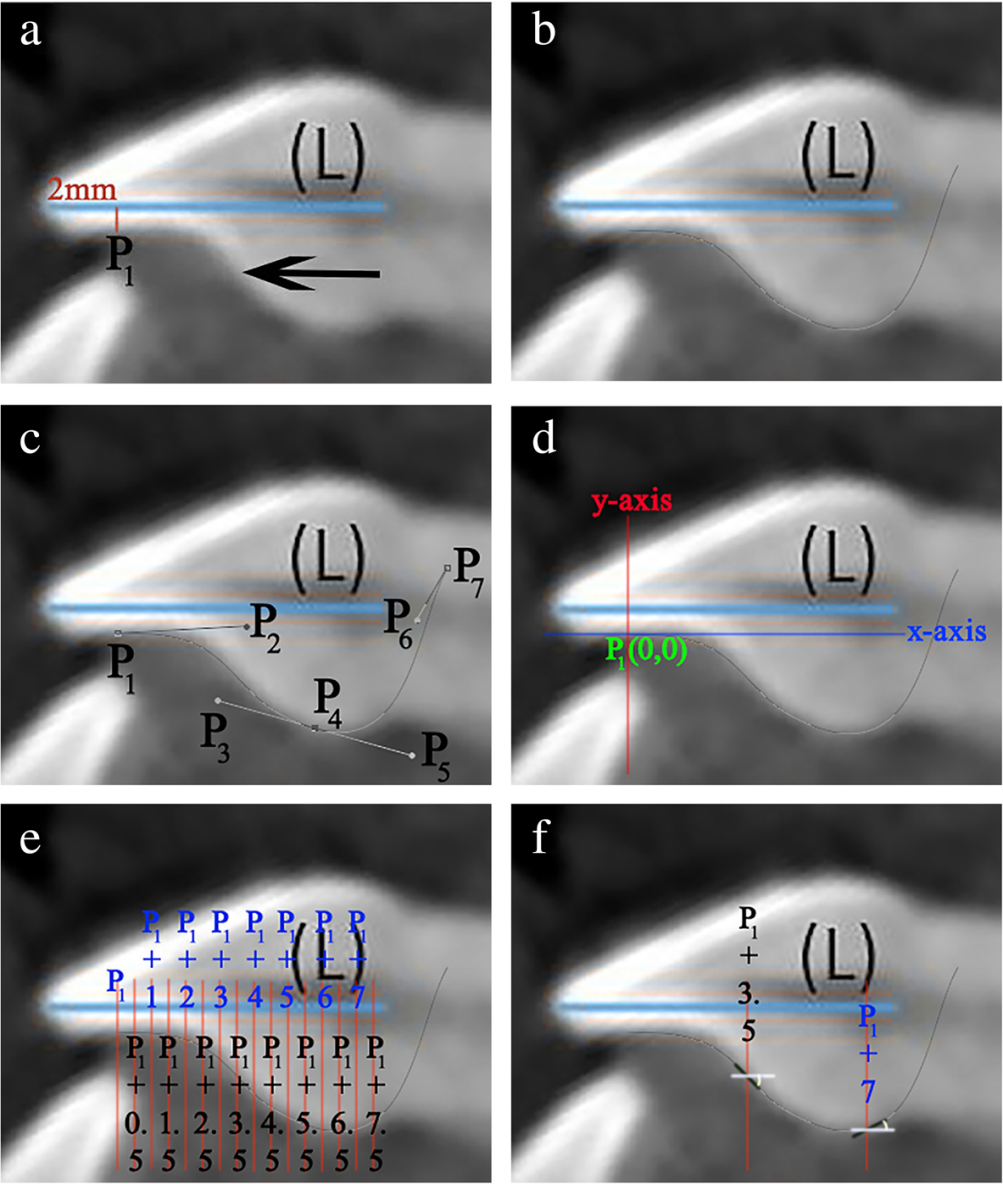
**a** Image shows crown’s long axis (L) and its perpendicular at 2 mm (red line), point P_1_ (intersection of red line with PSC) and the visually estimated location of inflection point. **b** Tracing of the first and second cubic poly-Bezier curves. **c** First cubic poly-Bezier extends from its initial anchor point P_1_ to its terminal anchor point P_4_, which in this case is located 2.1 mm cervical to the estimated visual position of inflection point. P_2_ and P_3_ are respectively the second and third control points of the first cubic poly-Bezier. Tracing of second cubic poly-Bezier extends from its initial anchor point P_4_ to its terminal anchor point P_7_ which was placed cervical and labial to palatal CEJ. The second control point (P_5_) of the second cubic poly-Bezier was positioned so both lengths and slopes of P_3_P_4_ and P_4_P_5_ are equal. P_6_ is the third control point of second cubic poly-Bezier. **d** Origin (P_1_) and *x*–y axes of the poly-Bezier curves. **e** Tangents to PSC are calculated at intersections of 0.5-mm increment lines with first and second cubic poly-Bezier curves. **f** Two examples of the tangents to PSC and their respective torque angles at [P_1_ + 3.5] and [P_1_ + 7]. The torque angle value is negative on [P_1_ + 3.5] and positive at [P_1_ + 7]

**Fig. 3:**
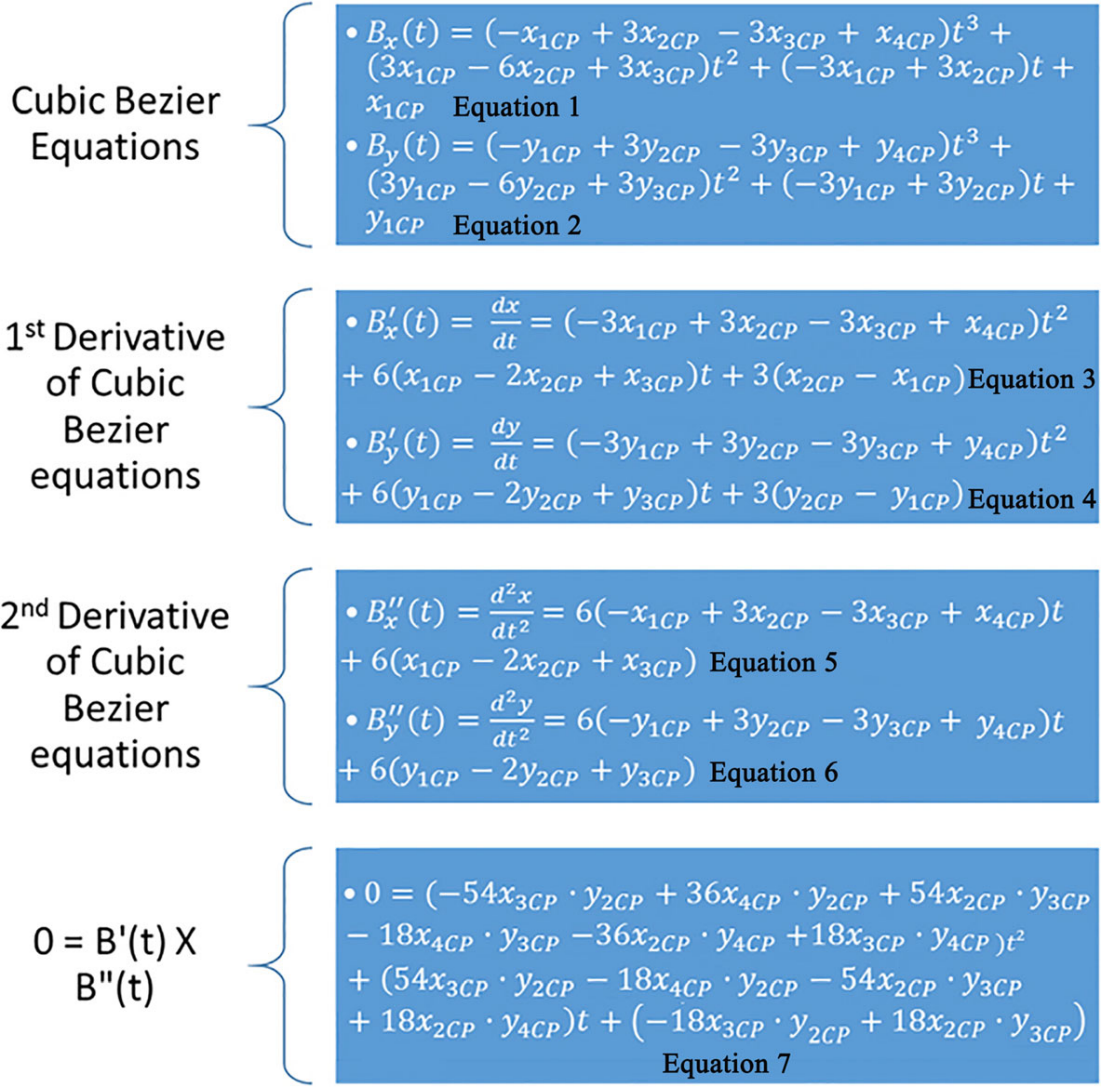
x_1CP_, x_2CP_, x_3CP_, and x_4CP_stand respectively for the *x* coordinate value of the first, second, third, and fourth control points of the respective cubic poly-Bezier curve (first or second). y_1CP_, y_2CP_, y_3CP_, and y_4CP_ stand respectively for the *y* coordinate value of the first, second, third, and fourth control points of the respective cubic poly-Bezier curve. The parameter *t* in all the equations below lies between 0 ≤ *t* ≤ 1. The first and second equations are the equations of the *x* and *y* components of the cubic Bezier curve respectively, while the third and fourth equations are their first derivatives, respectively, and the fifth and sixth equations are the second derivatives. The position of the inflection points of a parametric cubic Bezier curve are among the solutions of the equation: B^′^(*t*) *X* B^″^(*t*) = B′_*x*_(*t*) • B″_*y*_(*t*) ‐ B′_*y*_(*t*) • B″_*x*_(*t*), where B′(*t*) and B″(*t*) stand for the first and second derivative vectors, respectively, of Bezier curve and *X* stands for the cross product between the two vectors. It should be noted that Eq. 7 was written under these two considerations: the inflection point of PSC in this study was always located in the first cubic poly-Bezier and P_1_, the first control point of the first cubic poly-Bezier, has an *x* and *y* coordinate equal to zero (any term multiplied by zero is eliminated)

**Fig. 4:**
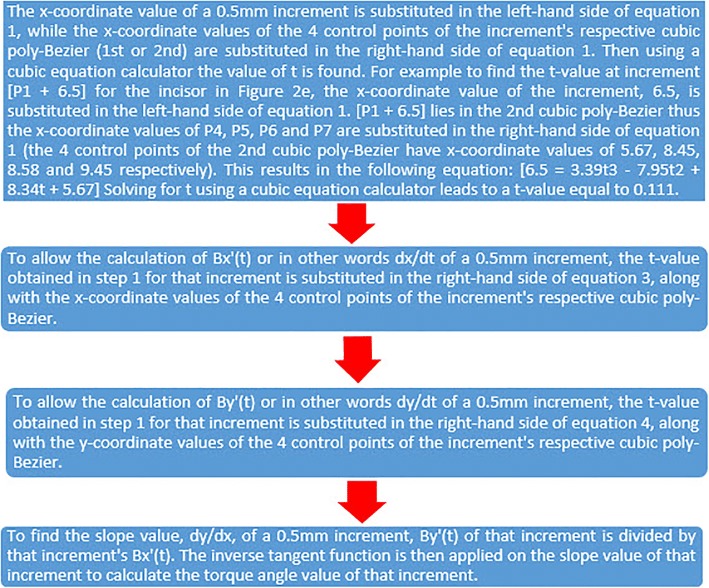
The procedures done to calculate the torque angle value at any 0.5-mm increment are described here in four steps. All the equations mentioned here are found in Fig. 3 and a specially formed Excel spreadsheet containing all these equations in addition to cubic and quadratic equation calculators, allowed all the procedures listed here to be done

**Fig. 5:**
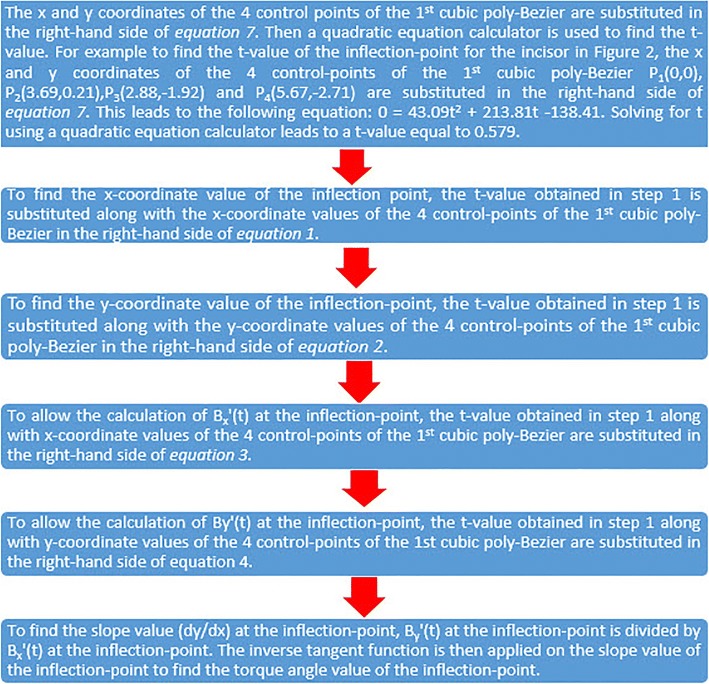
This figure lists in six steps the procedures that were done to calculate the exact location of the inflection point on PSC as well as its torque angle value. All the equations mentioned here are found in Fig. 3 and the specially formed Excel spreadsheet mentioned in the legend of Fig. 4 allowed all the procedures listed here to be done

The manipulation of each RMCI followed the procedures detailed below (all procedures were made by one orthodontist):

1. Using CS 3D Imaging Software 3.1.9 (Carestream Health, Rochester, NY), the axial slice in “Oblique Slicing” tab was selected and the indicator which represented the sagittal plane was oriented with the labio-palatal axis of the RMCI (Fig. 1a). In the sagittal slice, a line with a known measurement (calibration line) was drawn that was used later for calibration (Fig. 1b). A screenshot image of the workspace at double magnification was made and then the TIFF image was opened with Photoshop.
2. In Photoshop, the scale from pixels to millimeter was calibrated using the calibration line. The long axis of the crown was then drawn in Photoshop (Fig. 1c). The long axis of the crown was defined similarly to Bryant et al. [4] and van Loenen et al. [9] as a line drawn from the incisal edge of the incisor to the midpoint of the line joining the palatal and labial CEJ. The image was then rotated until the long axis of the crown became horizontal (parallel to Photoshop’s *x*-axis) (Fig. 1d). This rotation allows superimposition of all incisors on their crown’s long axis, enabling direct comparison of the torque angles.
3. As a single cubic Bezier curve failed in accurately describing the PSC, 2 cubic poly-Bezier curves were used. On the crown’s long axis, at a distance of 2 mm from the incisal edge, a line perpendicular to the crown’s long axis was drawn that intersected PSC at point P_1_ (Fig. 2a). The initial anchor point (first control point) of the first cubic poly-Bezier curve of PSC was P_1_, while its terminal anchor point (fourth control point) was point P_4_, a point located 1.5 to 3 mm cervical to the visually estimated position of the inflection point of PSC (Fig. 2a–c). Choosing the terminal anchor point of the first cubic poly-Bezier curve as previously described ensures that the inflection point of PSC is contained in the first cubic poly-Bezier and gives the ability to objectively determine the true position of inflection point on PSC as well as its ${Torque}_{Value}^{Angle}$ through accurate mathematical procedures.
4. To allow the first and second cubic poly-Bezier curves to be continuous and differentiable at P_4_, the second control point (P_5_) of the second cubic poly-Bezier was positioned so that line P_3_P_4_ and line P_4_P_5_ have equal lengths and slopes (Fig. 2c) [16]. The terminal anchor point of the second cubic poly-Bezier (P_7_) was placed in a position cervical and labial to the palatal CEJ (Fig. 2c), in order to allow the second cubic poly-Bezier to more accurately trace the part of PSC cervical to P_4_.
5. The origin of the 2 cubic poly-Bezier curves was set at P_1_, with the *x*-axis parallel to the long axis of the crown and the *y*-axis perpendicular to the *x*-axis (Fig. 2d). The *x*-axis and *y*-axis were positive in the right and upwards directions, respectively. The coordinates of the four control points that are needed to obtain the equations of each of the 2 cubic poly-Bezier curves were found using the ruler tool in Photoshop. Figure 3 gives the cubic Bezier curve formulas and the formulas of their first and second derivatives [15] that were used to find the slope at inflection point and at the 0.5-mm increments from P_1_ to palatal CEJ (Fig. 2e, f). Figures 4 and 5 list the procedures done in Microsoft Excel 2013 (Microsoft, Redmond, Washington) to calculate the ${Torque}_{Values}^{Angle}$ at the 0.5-mm increments and inflection point.
6. After calculating the ${Torque}_{Values}^{Angle}$ of all the 0.5-mm increments and inflection point, the incisor was divided into two incisor parts, a part incisal to the inflection point and a second part cervical to it. Incremental subtractions in each incisor part were done in Excel, where an incremental subtraction was defined as follows: the difference in the ${Torque}_{Value}^{Angle}$ between two successive 0.5 mm increments, where the more incisal increment was always subtracted from the more cervical increment (Table 1).

**Table 1:** The torque angle values at the 0.5-mm increments and at the inflection point of the incisor in Figs. 1 and 2 are shown in this table. As the palatal CEJ of this incisor is located at the *x* coordinate point 7.70, the most cervical 0.5-mm increment is [P_1_ + 7.50]. Incremental subtractions were calculated for the successive 0.5-mm increments in the incisor part incisal to the inflection point and in the incisor part cervical to the inflection point. An incisor part containing an *X* number of 0.5-mm increments contains a number equal to *X* − 1 of incremental subtractions. Note the progressive decrease in torque angle values of the increments from P_1_ to inflection point and the opposite progressive increase in the torque angle values of the increments from inflection point to the most cervical calculated increment. An incremental subtraction had a negative value in the incisor part incisal to inflection point and a positive value in the incisor part cervical to it. The inflection point had the most negative torque angle value on PSC

	0.5 mm increment	Torque angle value	Incremental subtraction	Number of incremental subtractions
Incisor part incisal to inflection point	[P_1_]	3.26		6
	[P_1_ + 0.50]	− 0.10	− 3.36	
	[P_1_ + 1.00]	− 4.61	− 4.51	
	[P_1_ + 1.50]	− 10.85	− 6.24	
	[P_1_ + 2.00]	− 19.53	− 8.68	
	[P_1_ + 2.50]	− 30.86	− 11.33	
	[P_1_ + 3.00]	− 42.21	− 11.35	
	Inflection point [P_1_ + 3.46]	− 46.67		
Incisor part cervical to inflection point	[P_1_ + 3.50]	− 46.63		8
	[P_1_ + 4.00]	− 41.54	5.09	
	[P_1_ + 4.50]	− 32.68	8.86	
	[P_1_ + 5.00]	− 24.37	8.31	
	[P_1_ + 5.50]	− 17.71	6.66	
	[P_1_ + 6.00]	− 9.24	8.47	
	[P_1_ + 6.50]	4.02	13.26	
	[P_1_ + 7.00]	21.41	17.39	
	[P_1_ + 7.50]	40.84	19.43	

### Statistical analysis

The statistical analysis was performed using the Statistical Package for Social Sciences SPSS (IBM SPSS Statistics version 23, Armonk, NY). Intra-observer reliability in tracing the RMCI and in calculating the ${Torque}_{Values}^{Angle}$ of their increments was determined using the Dahlberg formula, by randomly selecting 10 incisors and repeating the tracing and measuring procedures after 1 month. Descriptive statistics for the ${Torque}_{Values}^{Angle}$ of the 50 RMCI at the inflection point and at the 0.5-mm increments between [P_1_] and palatal CEJ were calculated. The frequency of positive and negative incremental subtractions in each incisor part of the 50 incisors was found. Since the data did not violate assumption of normality as detected by Shapiro-wilk test, one-way ANOVA was done to detect systematic differences between the mean ${Torque}_{Values}^{Angle}$ of the increments and when significant differences exist Tukey test was used post-hoc. The level of significance was set at *p* < 0.05 for all statistical tests.

## Results

The Dahlberg error for repetitive tracing and measuring procedures was 1.18°.

The mean ${Torque}_{Values}^{Angle}$ at all 0.5-mm increments and at inflection point showed high-standard deviations and a wide range of values for the 50 RMCI (Table 2).

**Table 2:** Descriptive statistics of the torque angle values of the PSC of the 50 incisors from [P_1_] to [P_1_ + 11] and at the inflection point. Descriptive statistics of the inflection point location on the PSC of the 50 studied incisors is also shown in this table. N stands for frequency of recorded site and SD for standard deviation

Site	N	Minimum	Maximum	Range	Mean	SD
[P_1_]	50	− 3.30	28.62	31.92	9.15	7.90
[P_1_ + 0.5]	50	− 7.62	19.25	26.87	4.95	6.32
[P_1_ + 1]	50	− 9.63	11.66	21.28	0.04	5.40
[P_1_ + 1.5]	50	− 18.38	3.42	21.80	− 6.10	5.38
[P_1_ + 2]	50	− 32.14	− 2.59	29.55	− 14.07	6.70
[P_1_ + 2.5]	50	− 44.96	− 7.59	37.37	− 24.44	9.40
[P_1_ + 3]	50	− 48.39	− 11.80	36.59	− 31.88	9.30
[P_1_ + 3.5]	50	− 60.77	− 16.91	43.86	− 36.34	8.79
[P_1_ + 4]	50	− 52.64	− 20.16	32.48	− 35.84	6.61
[P_1_ + 4.5]	50	− 45.96	− 18.24	27.73	− 33.31	6.22
[P_1_ + 5]	50	− 43.15	− 15.51	27.64	− 29.75	6.21
[P_1_ + 5.5]	50	− 50.27	− 9.85	40.42	− 26.04	7.47
[P_1_ + 6]	50	− 35.48	6.28	41.77	− 20.62	8.87
[P_1_ + 6.5]	50	− 31.96	7.64	39.60	− 14.00	9.84
[P_1_ + 7]	50	− 27.82	21.41	49.23	− 7.33	11.73
[P_1_ + 7.5]	50	− 20.81	40.84	61.65	0.10	13.77
[P_1_ + 8]	45	−14.02	48.09	62.11	9.18	15.83
[P_1_ + 8.5]	34	− 9.88	50.10	59.98	13.32	14.86
[P_1_ + 9]	25	− 5.31	46.92	52.24	16.69	12.74
[P_1_ + 9.5]	12	− 1.69	37.92	39.61	18.09	11.92
[P_1_ + 10]	4	4.91	34.30	29.39	20.77	12.07
[P_1_ + 10.5]	3	14.50	32.31	17.81	24.63	9.16
[P_1_ + 11]	1	32.92	32.92	0.00	32.92	–
Inflection point	50	− 70.84	− 22.71	48.13	− 45.82	9.98
Inflection point location	–	[P_1_ + 2.03]	[P_1_ + 6.17]	4.14	[P_1_ + 3.73]	0.94

All 50 incisors studied showed that their 0.5-mm increments had a progressive decrease in ${Torque}_{Values}^{Angle}$ from P_1_ to inflection point and an opposite progressive increase in ${Torque}_{Values}^{Angle}$ from inflection point to the most cervical calculated increment, with the inflection point exhibiting the most negative ${Torque}_{Value}^{Angle}$. All incremental subtractions in the incisor part cervical to inflection point were positive (478 incremental subtractions), and all incremental subtractions in the incisor part incisal to inflection point were negative (350 incremental subtractions) (Additional file 1). 

The one-way ANOVA test showed a highly statistically significant difference between the increments, F(22,885) = 137.60, *p* < 0.0001 (Table 3). The results of the post-hoc Tukey test were mostly significant and are presented in Table 4.

**Table 3:** One-way ANOVA test shows a highly significant difference (*p* < 0.0001) between the torque angle values of the increments

	Sum of squares	df	Mean square	F	Significance
Between groups	270,831.54	22	12,310.52	137.60	< 0.0001
Within groups	79,176.18	885	89.46		
Total	350,007.72	907	385.90

**Table 4:** Post-hoc Tukey test summarized and presented in the table below. The left side of the table shows an increment while the right side shows which increments were statistically significant different (*p* < 0.05) from that increment

Increment	Increments with a statistically significant difference
[P_1_]	[P_1_ + 1] to [P_1_ + 7.5]
[P_1_ + 0.5]	[P_1_ + 1.5] to [P_1_ + 7] and [P_1_ + 8.5] to [P_1_ + 9.5]
[P_1_ + 1]	[P_1_], [P_1_ + 2] to [P_1_ + 7] and [P_1_ + 8] to [P_1_ + 10.5]
[P_1_ + 1.5]	[P_1_] to [P_1_ + 0.5], [P_1_ + 2] to [P_1_ + 6.5] and [P_1_ + 8] to [P_1_ + 11]
[P_1_ + 2]	[P_1_] to [P_1_ + 5.5] and [P_1_ + 7] to [P_1_ + 11]
[P_1_ + 2.5]	[P_1_] to [P_1_ + 4.5] and [P_1_ + 6.5] to [P_1_ + 11]
[P_1_ + 3]	[P_1_] to [P_1_ + 2.5] and [P_1_ + 6] to [P_1_ + 11]
[P_1_ + 3.5]	[P_1_] to [P_1_ + 2.5] and [P_1_ + 5.5] to [P_1_ + 11]
[P_1_ + 4]	[P_1_] to [P_1_ + 2.5] and [P_1_ + 5.5] to [P_1_ + 11]
[P_1_ + 4.5]	[P_1_] to [P_1_ + 2.5] and [P_1_ + 5.5] to [P_1_ + 11]
[P_1_ + 5]	[P_1_] to [P_1_ + 2] and [P_1_ + 6] to [P_1_ + 11]
[P_1_ + 5.5]	[P_1_] to [P_1_ + 2], [P_1_ + 3.5] to [P_1_ + 4.5] and [P_1_ + 6.5] to [P_1_ + 11]
[P_1_ + 6]	[P_1_] to [P_1_ + 1.5], [P_1_ + 3.0] to [P_1_ + 5] and [P_1_ + 7] to [P_1_ + 11]
[P_1_ + 6.5]	[P_1_] to [P_1_ + 1.5], [P_1_ + 2.5] to [P_1_ + 5.5] and [P_1_ + 7.5] to [P_1_ + 11]
[P_1_ + 7]	[P_1_] to [P_1_ + 1],[P_1_ + 2.0] to [P_1_ + 6] and [P_1_ + 7.5] to [P_1_ + 11]
[P_1_ + 7.5]	[P_1_], [P_1_ + 2] to [P_1_ + 10.5]
[P_1_ + 8]	[P_1_ + 1] to [P_1_ + 7.5]
[P_1_ + 8.5]	[P_1_ + 0.5] to [P_1_ + 7.5]
[P_1_ + 9]	[P_1_ + 0.5] to [P_1_ + 7.5]
[P_1_ + 9.5]	[P_1_ + 0.5] to [P_1_ + 7.5]
[P_1_ + 10]	[P_1_ + 1] to [P_1_ + 7.5]
[P_1_ + 10.5]	[P_1_ + 1] to [P_1_ + 7.5]
[P_1_ + 11]	[P_1_ + 1.5] to [P_1_ + 7]

## Discussion

**Fig. 6:**
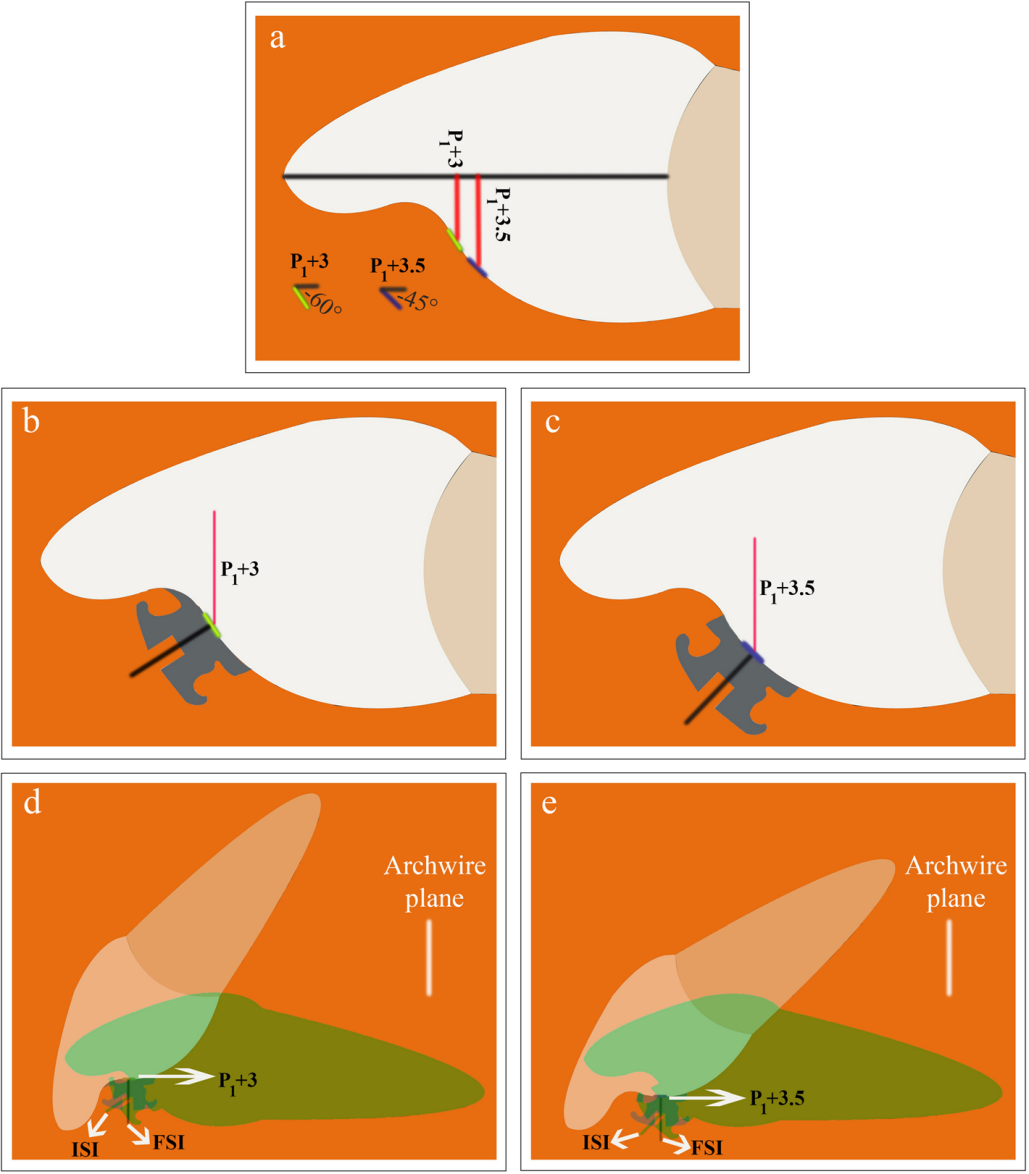
**a** Increments [P_1_ + 3] and [P_1_ + 3.5] has a ${Torque}_{Value}^{Angle}$ equal to − 60° and − 45°, respectively. The incremental subtraction value between [P_1_ + 3.5] and [P_1_ + 3] is + 15°. **b**, **c** When a bracket with a 0° third order prescription is placed at [P_1_ + 3] or [P_1_ + 3.5], the slot inclination of the bracket will be perpendicular to the tangent at [P_1_ + 3] or [P_1_ + 3.5], respectively. The + 15° difference in incremental subtraction value between [P_1_ + 3.5] and [P_1_ + 3] is reflected as a 15° difference in the slot inclination. **d**, **e** Placement of a full-sized rectangular wire with minimal play in the slot of the bracket in **b** or in **c**, will change the initial slot inclination (ISI) to a final slot inclination (FSI) parallel to the archwire plane. The horizontally positioned incisor in **d**, **e** is the initial position of the incisor before rectangular wire placement, while the incisor superimposed on it represents its new position after the rectangular wire placement. Cervically shifting the vertical bracket position from [P_1_ + 3.0] as in **b** and **d** to [P_1_ + 3.5] as in **c** and **e**, results in a 15° of decreased crown palatal/root labial incisor movement in **e** compared to **d**. The positive incremental subtraction value between [P_1_ + 3.5] and [P_1_ + 3] resulted in an extra + 15° of crown labial/root palatal incisor movement in **e** compared to **d**

The inflection point of PSC of maxillary central incisor has utmost importance in understanding the directional change in torque which will occur upon a 0.5-mm shift in a vertical bracket position. As incremental subtractions were always positive cervical to inflection point and negative incisal to it, the following can be derived: Cervically oriented shifts or errors in vertical bracket position in an RMCI cause crown labial/root palatal torque changes cervical to inflection point (Fig. 6) and crown palatal/root labial torque changes incisal to it, while incisally oriented shifts cause movements opposite to the mentioned ones cervical and incisal to inflection point, respectively. Furthermore, the more cervical a bracket is placed on the incisor part cervical to inflection point, the more is the potential of crown labial/root palatal torque expression. Conversely, the more cervical a bracket is placed on the incisor part incisal to inflection point, the more is the potential of crown palatal/root labial torque expression. As the inflection point exhibits the most negative ${Torque}_{Value}^{Angle}$ on PSC, it is the site with most crown palatal/root labial torque expression potential. The characteristics of the inflection point mentioned in the four previous sentences justify its use as a torque reference for lingual bracket positioning on the PSC of the maxillary central incisor. Bracket position on PSC specified as either incisal or cervical to inflection point allows the orthodontist to recognize the inherent characteristics of the bracket site rather than it being specified arbitrary and thus devoid of this recognition.

The vast extent of ${Torque}_{Values}^{Angle}$ at all the 0.5-mm increments of the 50 RMCI studied (Table 2) and the statistically significant differences between the ${Torque}_{Values}^{Angle}$ of the increments (Tables 3 and 4) are a scientific justification through a mathematical model (cubic Bezier) for the use of customized bracket torque prescription on PSC. The adoption of a pre-established bracket torque prescription is inappropriate for embracing the extremely varying PSC morphology of the maxillary central incisor. The most common maxillary central incisor lingual bracket torque prescriptions of 40°, 55°, and 68° are not sufficient to cover the wide spectrum of ${Torque}_{Values}^{Angle}$ at each of the 0.5-mm increments.

**Fig. 7:**
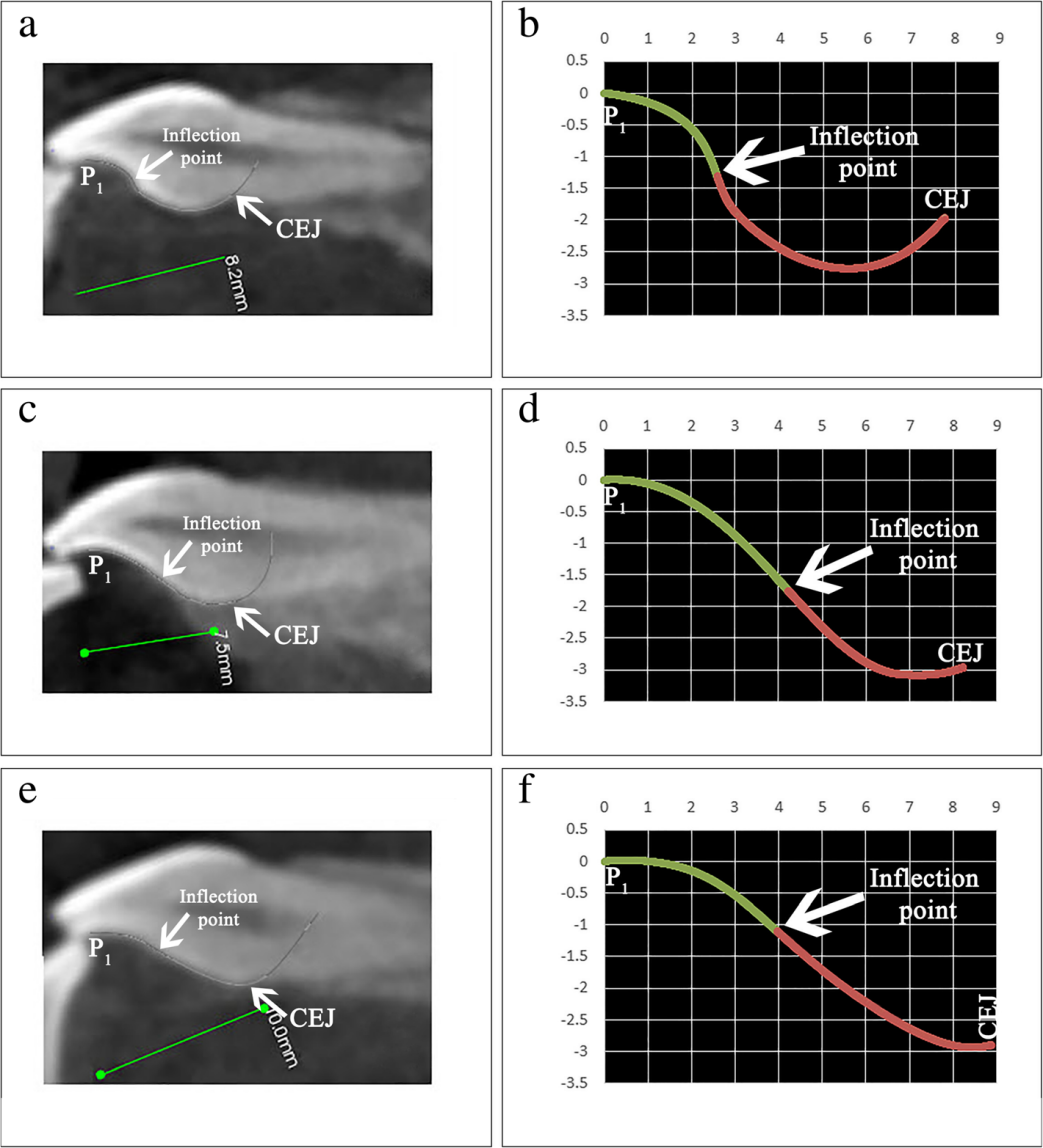
**a**, **c**, **e** Three incisors with a complex, moderate, and simple S-shaped PSC, respectively, while **b**, **d**, **f** show their PSC plotted in Excel using cubic Bezier equations of Table 1

This study is in agreement with other studies that reported on the wide variability in PSC morphology [1, 2, 4]. The PSC form of the incisors in this study varied from slight to moderate to complex S-shaped curvatures (Fig. 7). The aforementioned difference in PSC form justifies the approach in lingual orthodontics to individualize the base of each maxillary central incisor lingual bracket [18]. The wide range in the forms of PSC is responsible for the broad variation in the ${Torque}_{Values}^{Angle}$ of the 0.5-mm increments. Also contributing to this broad variation of ${Torque}_{Values}^{Angle}$ is the anatomical location of the 0.5-mm increment being measured. For example, the increments [P_1_ + 3] and [P_1_ + 3.5] of the RMCI in Fig. 7b are cervical to the inflection point while in the incisor of Fig. 7d those two increments are incisal to it. The anatomical variation in increment location with respect to the inflection point between incisors leads to distinctly different ${Torque}_{Values}^{Angle}$.

## Conclusions

1. The inflection point is the anatomical landmark on PSC where directional change in torque occurs in a maxillary central incisor, as of this it can be used as a torque reference for lingual bracket positioning on PSC.
2. Cervically oriented shifts in vertical bracket position in an RMCI cause crown palatal/root labial torque changes incisal to inflection point and crown labial/root palatal torque changes cervical to inflection point, while incisally oriented shifts cause opposite movements incisal and cervical to inflection point, respectively.
3. The high-standard deviation of the mean ${Torque}_{Values}^{Angle}$ of all the 0.5-mm incremental PSC sites of the 50 studied incisors calls for the fabrication of customized brackets that incorporates individualized torque prescriptions appropriate to vertical bracket position.

## Additional file

Additional file 1: The torque angle value of each 0.5 mm increment and of each inflection point for each of the 50 RMCI used in this study are found in this additional file. Furthermore, the location of each inflection point of each of the 50 RMCI is disclosed here. Each value for each incremental subtraction in the incisor part incisal to inflection point or in the incisor part cervical to the inflection point for each RMCI is also shown in this additional file. (XLSX 76 kb)

## Abbreviations

CBCT: Cone beam computed tomography
PSC: Palatal surface curvature
RMCI: Right maxillary central incisor
